# Comparison of the effects of spinal anesthesia, paracervical block and general anesthesia on pain, nausea and vomiting, and analgesic requirements in diagnostic hysteroscopy: A non-randomized clinical trial

**DOI:** 10.3389/fmed.2023.1089497

**Published:** 2023-03-01

**Authors:** Nahid Manouchehrian, Shamim Pilehvari, Farshid Rahimi-Bashar, Farzaneh Esna-Ashari, Shaghayegh Mohammadi

**Affiliations:** ^1^Department of Anesthesiology, Fatemi Medical Center, Hamadan University of Medical Sciences, Hamedan, Iran; ^2^Department of Gynecology, Fatemi Medical Center, Hamadan University of Medical Sciences, Hamedan, Iran; ^3^Department of Anesthesiology, Hamadan University of Medical Sciences, Hamedan, Iran; ^4^Department of Community Medicine, Medical Sciences Faculty, Hamadan University of Medical Sciences, Hamedan, Iran; ^5^Medical Sciences Faculty, Hamadan University of Medical Sciences, Hamedan, Iran

**Keywords:** hysteroscopy, anesthesia, paracervical, general anesthesia, spinal anesthesia

## Abstract

**Background:**

The aim of this study was to compare the effect of spinal anesthesia (SPA), paracervical block (PB), and general anesthesia (GA), on pain, the frequency of nausea and vomiting and analgesic requirements in diagnostic hysteroscopy.

**Methods:**

This single-center, non-randomized, parallel-group, clinical trial was conducted on 66 diagnostic hysteroscopy candidates who were selected by convenience sampling at Fatemieh Hospital, in Hamadan, Iran, in 2021.

**Results:**

The mean pain score during recovery and the need for analgesic injections was found to be significantly higher in the GA group compared to that in the SPA group (pain: 3.77 ± 2.25 vs. 0.10 ± 0.30, *P* < 0.001), (analgesic: 50 vs. 0%, *P* < 0.001) and PB group (pain: 3.77 ± 2.25 vs. 0.90 ± 1.37, *P* < 0.001), (analgesic 50 vs. 10%, *P* < 0.001), respectively. However, no statistically significant difference was observed between the mean pain score between SPA and PB groups (0.10 ± 0.30 vs. 0.90 ± 1.3, *P* = 0.661). In addition, there were no significant differences between groups on nausea/vomiting after operation (*P* = 0.382). In adjusted regression analysis (adjusting for age, weight, gravid, abortion, and cause of hysteroscopy), the odds ratio (OR) of pain score during recovery was increased in PB (OR: 4.471, 95% CI: 1.527–6.156, *P* = 0.018) and GA (OR: 8.406, 95% CI: 2.421–9.195, *P* = 0.001) groups compared with the SPA group. However, in adjusting based on times of surgery duration, anesthesia duration, recovery and return of motor function, the ORs of pain score between groups was not statistically significant.

**Conclusion:**

Despite reduced pain during recovery in patients receiving SPA, duration of anesthesia, recovery period, and return of motor function were significantly prolonged compared to those receiving PB or GA. It seems that PB with less recovery time and faster return of motor function than SPA and also mild pain during recovery compared to GA can be a good option for hysteroscopy.

**Clinical trial registration:**

http://www.irct.ir, identifier IRCT20120915010841N26.

## Introduction

Hysteroscopy is a common endoscopic procedure used on an outpatient basis to diagnose and treat intrauterine pathologies (1, 2). The rate of recovery in outpatient surgery is one of the most important factors related to the patient’s discharge, which largely depends on the method of anesthesia (3). Hysteroscopy can be performed under general anesthesia (GA) (intravenous and inhalation), regional anesthesia (spinal and epidural), and local anesthesia (paracervical and intracervical block injections) (4–8). Choosing the appropriate anesthesia method for outpatient surgeries such as diagnostic hysteroscopy should be based on performing it in the shortest possible time, maximum control of pain during and post-surgery and minimum complications in the fastest recovery time (9). Complications such as pain, shivering, vertigo, nausea, and vomiting may delay the patient’s discharge or increase the likelihood of re-admission (10, 11).

Evidence shows that each method of anesthesia has its advantages and disadvantages, so to date no ideal anesthesia method has been proposed for hysteroscopy surgery and there is definitely a need for further study in this area (12, 13). Rapid patient recovery, cost-effectiveness, anesthesiologists’ familiarity, and preference for anxious patients, has made GA suitable for a variety of outpatient surgeries, including diagnostic hysteroscopy (14, 15). However, pain, nausea/vomiting, shortness of breath, hemodynamic abnormalities, vertigo, persistent effects of sedatives, aspiration pneumonia and gastrointestinal symptoms are the most common complications of GA (16, 17). Regional anesthesia, including spinal anesthesia (SPA) and epidural anesthesia (EDA), has fewer complications (pain, nausea and vomiting) than GA (6, 7, 18). However, failed SPA can cause hypotension, bradycardia, post-dural puncture headache (PDPH), urinary retention and sometimes neurological complications (19–22). On the other hand, paracervical block (PB) is commonly used as local anesthesia method in pain reduction during cervical dilatation and uterine interventions (23, 24). However, limited duration of anesthesia, procedural pain, respiratory depression, excessive sedation, hypotension and bradycardia are common complications of PB (25–27).

Despite the fact that many studies have been conducted regarding the safest and most time and cost-effective way for pain reduction in diagnostic hysteroscopy (28, 29) no study has been performed comparing all three anesthesia methods and there is still controversy on the best procedure. The purpose of this non-randomized clinical trial was to evaluate the efficacy and effects of GA, SPA, and PB on pain, nausea, vomiting, and analgesic requirements among patients undergoing diagnostic hysteroscopy.

## Materials and methods

### Trial design

The study was designed as a single-center, non-randomized, parallel-group, clinical trial in accordance with Consolidated Standards of Reporting Trials (CONSORT) guidelines (CONSORT checklist as Supplementary material is available) (30). Ethics approval was obtained from the Ethics Committees of Hamadan University of Medical Sciences (IR.UMSHA.REC.1399.875). The study was registered in Iranian Registry of Clinical Trial^1^ by the number of (IRCT20120915010841N26). Written informed consent was obtained from each patient. This study was conducted in accordance with the World Medical Association Declaration of Helsinki: ethical principles for medical research involving human subjects (31).

### Study population and sample size

The participants were women between the ages of 18 and 45, who were candidates for diagnostic hysteroscopy under SPA, PB, and GA, referred to Fatemieh Hospital, Hamadan, Iran, in 2021. The sample size was estimated according to the results of a similar study (18), that showed significantly lower pain in the SPA group compared with GA at 12 h hysterectomy (μ1 for SPA group: 2.55 and sd 1:1.05) and (μ2 for GA group: 3.85 and sd 2:1.42). Considering a type I error (α) set as two-sided 5% (Z1-α/2 = 1.96), type II error (β) set as 20% (Z1-β = 0.84) and 80% study power, 22 people were estimated for each group. Eligible patients who met all inclusion criteria entered the study using a convenience sampling method, and convenience sampling continued until such time as the target sample size was achieved.


$$n=\frac{\left(s\,d\,1^{2}+s\,d\,2^{2}\right)+{\left(Z_{\alpha }+Z_{\beta }\right)}^{2}}{{\left(\mu \,1-\mu \,2\right)}^{2}}$$

Since anesthesia method must be selected in accordance with the patient’s wishes, the allocation of patients to the three study groups was non-random, and the study was conducted in a single-blind manner, with the patient and anesthesiologist aware of the type of anesthesia, and only data collectors and analyzers blinded.

The inclusion criteria included women aged 18–45 years, in grades I and II of American Society of Anesthesiologists (ASA) (32), candidate for diagnostic hysteroscopic surgery, no analgesic use for 24 h before the study, absence of any contraindications to SPA and GA, and willingness to participate in the study. On the other hand, the exclusion criteria were coagulation disorders, history of cardiovascular diseases, respiratory diseases, renal diseases, liver diseases, severe neurological disorders, Uterine prolapse, a history of allergies to local anesthetic and propofol, a history of previous cervical or hysteroscopic surgery, history of nausea and vomiting following previous anesthesia or history of motion sickness, a history of severe migraine headaches and a difficult airway, candidate patients for myomectomy or polypectomy with resectoscope, patients with electrolyte disturbances (sodium, potassium, and calcium) and lack of patient cooperation after initial interventions.

### Anesthesia procedure

Pre- anesthesia procedure: In general, 500 ml of normal saline was injected intravenously for all patients in each group within half an hour before the patient was transferred to the operating room.

Spinal Anesthesia (SPA) Procedure: In SPA group, fentanyl 1 mic/kg and midazolam 0.02 mg/kg were administered intravenously. Then SPA was performed with a 25-gauge Quincke spinal needle in a sitting position at L3–L4 or L4–L5 space by an experienced anesthesiologist (with more than 20 years of working in anesthesia), and 2.5 ml of bupivacaine 0.5% was injected into the subarachnoid space. Positive aspiration of clear cerebrospinal fluid before and after the injection confirmed correct needle placement. Then, the patient was placed in supine position and when the level of anesthesia reaches T10 area, surgery was allowed to begin.

Paracervical Block (PB) Procedure: In the PB group, 0.02 mg/kg midazolam and 1 mic/kg fentanyl were injected intravenously. After that, 10 ml of 2% lidocaine solution was injected by an experienced gynecologist (with more than 15 years of experience in gynecological surgery) at 3, 5, 7, and 9 o’clock position at the junction of cervix and vagina at an estimated depth of 1 cm using a 22-gauge spinal needle. The operation commenced after ensuring adequate anesthesia.

General Anesthesia (GA) Procedure: In the GA group, induction of anesthesia was carried out with 0.02 mg/kg of midazolam, 1 mic/kg fentanyl, 2 mg/kg of propofol, and 0.5 mg/kg atracurium then, trachea was intubated. Maintenance of anesthesia was performed with 50% N_2_O and O_2_ and also 1 MAC of isoflurane with controlled ventilation. At the end of the surgery, the residual of neuromuscular block was reversed with a mixture of 0.02 mg/kg body weight atropine and 0.04 mg/kg body weight of neostigmine and after making sure that the patient’s respiratory status was sufficient, the patient was extubated.

### Data collection

Baseline demographic and clinical data such as age, weight, the number of gravid, abortion and the cause of hysteroscopy were collected using a checklist specifically prepared for this study. Standard monitoring including electrocardiography, pulse oximetry and non-invasive blood pressure (NIBP) monitoring was performed for each patient. Hemodynamic parameters such as systolic blood pressure (SBP), diastolic blood pressure (DBP), mean arterial pressure (MAP), heart rate (HR), and oxygen saturation (SpO_2_) were monitored using X162 (Saadat Co., Iran). The hemodynamic parameters were recorded at seven different times (T1: pre-anesthesia, T2: during-anesthesia, T3: during-tenaculum, T4: during-cervical dilation, T5: during-hysteroscopy, T6: during-biopsy, and T7: during-hysteroscopy removal) for each patient. In addition to the dynamic parameters, during surgery, pain score (based on visual analog scale; VAS) (33), sedation level (assessed by Ramsay scale) (34), hypotension (SBP less than 90 mmHg), bradycardia (HR fewer than 60 beat per minute) were measured by two experienced anesthesia nurses, who were trained by an anesthesiologist before starting the study.

Episodes of hypotension (SBP less than 90 mmHg) and bradycardia (HR fewer than 60 beat per minute) were managed with intravenous ephedrine (10 mg) and atropine (0.5 mg), respectively. The total doses of ephedrine and atropine were also recorded. Shivering and intensity of shivering were evaluated by the bedside shivering assessment scale (BSAS) (35), and recorded for all groups.

Duration of anesthesia (from the time of injection of midazolam and fentanyl until the complete return of sensory-motor and level of consciousness) and surgery duration (after performing anesthesia and establishing the lithotomy position until removing the hysteroscope and returning the patient’s position to the supine position), duration of recovery period (from arriving to recovery room until the discharge time) and as well as the return time of motor functions (according to Bromage Scale) were recorded for each patient. After surgery, all patients were transferred to the recovery unit. In recovery, pain score (based on VAS) and sedation level (assessed by Ramsay scale) were measured in all patients and if the pain score of the patients was more than 3, Meperidine 0.5 mg/kg was injected intravenously and the amount and time of injection were recorded. Nausea and vomiting, shivering, vertigo, need for analgesic (need for intravenous injection of meperidine in case of pain score more than 3), time of administration of analgesic, return time of motor function (based on Bromage scale) (36), recovery time (based on Aldrete discharge criteria) (37, 38) and patient’s satisfaction were also assessed and recorded when patient’s level of sedation was back to normal (The descriptions for all measurement tools are available at, Supplementary Table 1).

### Statistical analysis

All analyses were conducted using SPSS software (ver.21) (SPSS Inc. Chicago, IL, USA) and in all analyses, a two-tailed *P*-value of < 0.05 was considered significant. Data normality was assessed with the Shapiro-Wilk Test. Descriptive statistics for continuous variables were presented as mean ± standard deviation (SD) for normal data and median and interquartile range (IQR) for non-normal data, with categorical variables presented as number/frequency (percentage). Demographics and clinical variables were compared between the three study groups using one-way analysis of variance (ANOVA) or Kruskal-Wallis H test for normally distributed, and non-normally distributed data, respectively, with *post-hoc* Bonferroni’s test for both. Chi-square or Fisher’s exact tests were used for categorical variables. Both unadjusted and adjusted (adjusting for age, weight, gravid, and abortion) repeated measures ANOVA (RMANOVA) were assessed, with *post-hoc* multiple Bonferroni’s tests to explore differences between pairwise groups. In addition, unadjusted and adjusted binary logistic regression analysis was performed to predict pain score for the three groups. We first adjusted the groups for age, weight, abortion, and cause of hysterectomy, and then based on surgery, anesthesia and recovery duration, as well as motor function return time. Associations in regression analysis were reported using the odds ratio (OR) and 95% confidence interval (CI). GraphPad Prism 9^©^ (GraphPad Software Inc., La Jolla, CA, USA) was used for forest plot of logistic regression analysis.

## Results

### Participant characteristics

**FIGURE 1:**
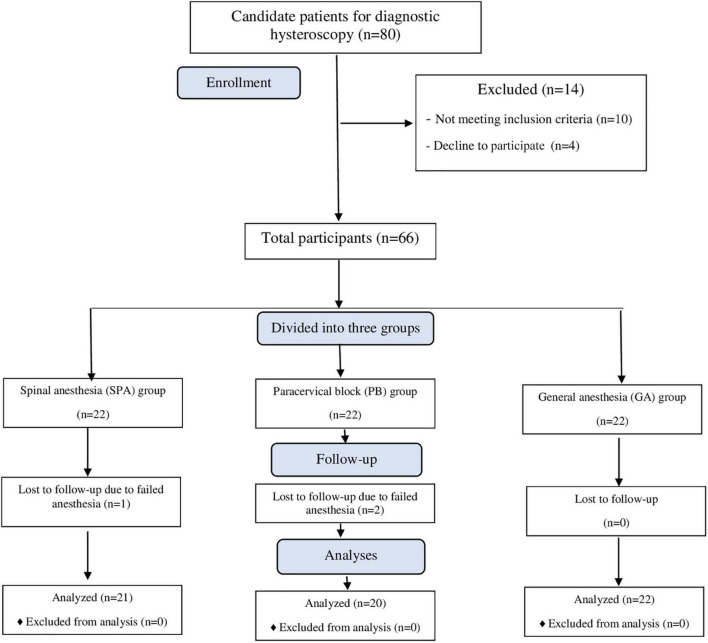
The CONSORT flow chart of study population.

Figure 1 shows the enrollment flow chart of participants. During the study, 80 patients underwent diagnostic hysteroscopy, of whom 66 were included in the study due to meeting all the inclusion criteria. In total, 66 participants were examined in three equal groups (*n* = 22) under GA, SPA, and PB. However, a patient in the SPA group and 2 patients in the PB group were excluded due to failed anesthesia, requiring a change to GA. After exclusions, 63 patients were included in the final analysis, comprising 21 patients in the SPA group, 20 patients in the PB group and 22 patients in the GA group.

### Comparison of demographic and clinical characteristics

Table 1 shows the comparison of demographic and clinical parameters between the three groups of study. There were no statistically significant differences in age (*P* = 0.283), weight (*P* = 0.851), gravid (*P* = 0.863), number of gravid (*P* = 0.935), abortion (*P* = 0.898), number of abortion (*P* = 0.578), cause of hysteroscopy (*P* = 0.209), ephedrine (*P* = 0.682), and atropine administered (*P* = 0.234) between the groups. While surgery duration was significantly shorter in the SPA group compared to PB and GA groups, anesthesia duration, return of motor function, and recovery time were all significantly prolonged. No differences were observed between PB and GA groups (*P* > 0.05).

**TABLE 1:** Comparison of demographic and clinical characteristics between three groups of study.

Variables		Groups (*n* = 63)			*P*-value
		SPA (*n* = 21)	PB (*n* = 20)	GA (*n* = 22)	
Age (years)	Mean ± SD	33.10 ± 5.06	35.05 ± 7.27	36.09 ± 6.11	0.283^a^
	(Range)	(26–40)	(21–44)	(21–46)	
Weight (kg)	Mean ± SD	68.85 ± 11.47	69.01 ± 8.66	67.45 ± 9.10	0.851^a^
	(Range)	(50–95)	(57–95)	(55–92)	
Gravid	No (%)	10 (47.6)	8 (40)	9 (40.9)	0.863^b^
	Yes (%)	11 (52.4)	12 (60)	13 (59.1)	
Number of gravidas	One (%)	5 (23.8)	4 (20)	6 (27.3))	0.935^b^
	>1 (%)	6 (28.6)	8 (40)	7 (31.8)	
Abortion	No (%)	17 (81)	15 (75)	17 (77.3)	0.898^b^
	Yes (%)	4 (19)	5 (25)	5 (22.7)	
Number of abortions	One (%)	1 (4.8)	4 (20)	3 (13.6)	0.578^b^
	>1 (%)	3 (14.3)	1 (5)	2 (9.1)	
Cause of hysteroscopy	Infertility (%)	10 (47.6)	12 (60)	13 (59.1)	0.209^b^
	Bleeding (%)	2 (9.5)	5 (25)	1 (4.5)	
	Post-pregnancy (%)	3 (14.3)	2 (10)	2 (9.1)	
	Polypectomy (%)	3 (14.3)	0	2 (9.1)	
	Myomectomy (%)	2 (9.5)	1 (5)	0	
	IUD removal (%)	0	0	3 (13.6)	
	Repeated abortion (%)	1 (4.8)	0	1 (4.5)	
**Times (min)**					
Surgery duration	Median (IQR)	30 (25–35)	40 (30–49)	45 (35–55)	**0.002** ^c^ *
Anesthesia duration	Median (IQR)	115 (105–147)	55 (50–65)	70 (55–80)	<**0.001**^c^*
Return time of motor function	Median (IQR)	70 (62–97)	5 (5–10)	15 (14–20)	<**0.001**^c^*
Recovery duration	Median (IQR)	80 (70–105)	35 (30–40)	50 (30–55)	<**0.001**^c^*
**Drug consumptions (%)**					
Ephedrine administered	Yes (10 mg)	3 (14.3)	0	1 (4.5)	0.157^b^
Atropine administered	Yes (0.5 mg)	0	4 (20)	2 (9.1)	0.092^b^

SPA, spinal anesthesia; PB, paracervical block; GA, general anesthesia. *Bold: *P*-value < 0.05 considered as significant.

^a^One-way ANOVA test.

^b^Chi-square test.

^c^Kruskal-Wallis test.

*Post-hoc* Bonferroni’s test showed a significant difference between SPA groups with both PB and GA groups at all times of surgery, anesthesia, return to motor function and recovery time. However, no significant differences were observed between PB and GA groups.

### Comparison of hemodynamics, sedation and VAS score pre and during surgery

**FIGURE 2:**
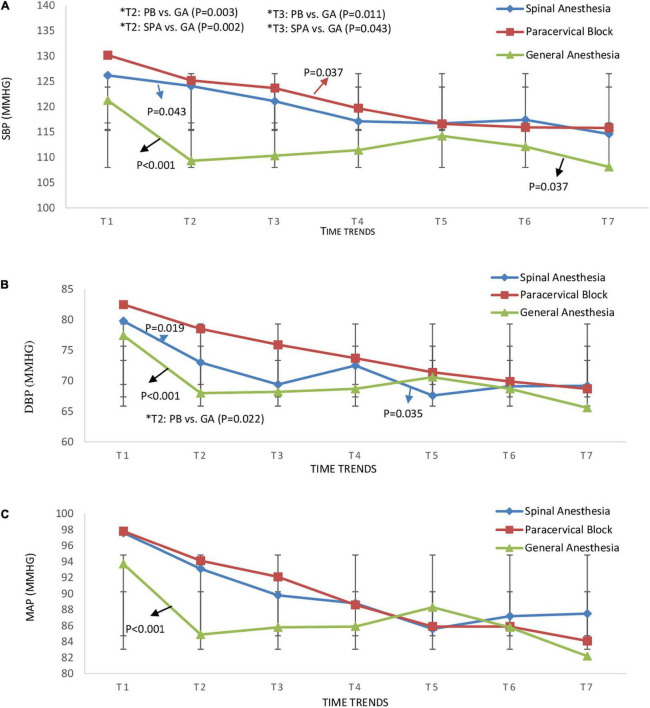
Time trend changes in panel **(A)** systolic blood pressure (SBP) (mmHg), **(B)** diastolic blood pressure (DBP) (mmHg), and **(C)** mean arterial pressure (MAP) (mmHg) in three groups of study. Time trends as follows; T1: pre-anesthesia, T2: during-anesthesia, T3: during-tenaculum, T4: during-cervical dilation, T5: during-hysteroscopy, T6: during-biopsy, and T7: during-hysteroscopy removal. The *P*-value within group differences are indicated by arrows and between groups by *. SPA, spinal anesthesia; PB, paracervical block; GA, general anesthesia.

**FIGURE 3:**
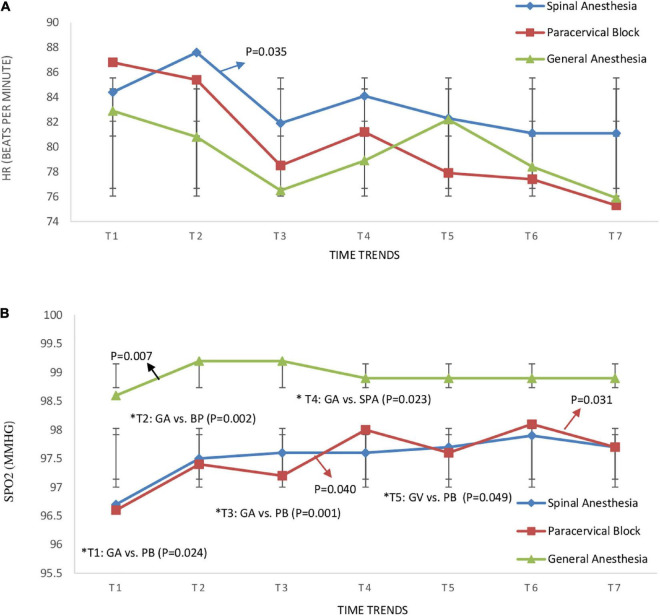
Time trend changes in panel **(A)** heart ratio (HR) (beats per minute) and **(B)** oxygen saturation (SPO_2_) (mmHg) in three groups of study. Time trends as follows; T1: pre-anesthesia, T2: during-anesthesia, T3: during-tenaculum, T4: during-cervical dilation, T5: during-hysteroscopy, T6: during-biopsy, and T7: during-hysteroscopy removal. The *P*-value within group differences are indicated by arrows and between groups by *. SPA, spinal anesthesia; PB, paracervical block; GA, general anesthesia.

The monitoring of the hemodynamic status in the three groups from pre-anesthesia until hysteroscopy removal (Table 2) showed that the SBP of the GA group at the time of anesthesia was significantly lower than that of the SPA group (109.27 ± 12.58 vs. 124.05 ± 15.72, *P* = 0.002) and PB group (109.27 ± 12.58 vs. 125.20 ± 14.15, *P* = 0.003) (Figure 2A). Similarly, the DBP of the GA group at this time was significantly lower than the PB group (68.00 ± 12.02 vs. 78.50 ± 12.52, *P* = 0.022) (Figure 2B). At the time of tenaculum insertion, the SBP of the GA group remained significantly lower than that of the PB group (110.32 ± 13.95 vs. 123.70 ± 15.69, *P* = 0.011) (Figure 2A). However, no significant differences were noted regarding MAP between the groups (Figure 2C). Time trend changes in HR ans SpO2 are shown in Figures 3A, B. The SpO_2_ of the GA group at the time of cervical dilation and hysteroscopy was significantly higher than that of the SPA group (98.95 ± 1.17 vs. 97.62 ± 2.20, *P* = 0.023) and PB group (98.91 ± 1.065 vs. 97.60 ± 1.84, *P* = 0.049), respectively (Figure 3B).

**TABLE 2:** Comparison of hemodynamic parameters, sedation level and VAS scores at different times between three groups of study.

Variables/Groups		Times, min (Mean ± SD)							*P*-value^b^	*P*-value^c^	*P*-value^e^
		Pre-anesthesia	During-anesthesia	During-tenaculum	During-cervical dilation	During-hysteroscopy	During-biopsy	During-hysteroscopy removal			
SBP (mmHg)	**SPA**	126.19 ± 13.37	124.05 ± 15.72	121.13 ± 13.39	117.14 ± 11.76	116.71 ± 11.01	117.48 ± 9.108	114.62 ± 8.26	<0.001*	0.023*	0.047*
	**PB**	130.20 ± 15.21	125.20 ± 14.15	123.70 ± 15.69	119.75 ± 16.37	116.65 ± 13.23	115.95 ± 14.58	115.80 ± 14.31	<0.001*	***P*** **-value** ^d^	***P*** **-value** ^f^
	**GA**	121.36 ± 13.67	109.27 ± 12.58	110.32 ± 13.95	111.45 ± 15.05	114.27 ± 15.54	112.05 ± 13.79	108.09 ± 9.62	<0.001*	<0.001*	0.076
*P*-value^a^		0.134	0.001*	0.008*	0.172	0.795	0.357	0.054			
DBP (mmHg)	**SPA**	79.81 ± 12.91	73.00 ± 12.26	69.43 ± 10.46	72.52 ± 14.21	67.62 ± 12.77	69.19 ± 10.16	69.24 ± 12.19	0.001*	0.348	0.075
	**PB**	82.55 ± 12.02	78.50 ± 12.52	75.95 ± 12.01	73.70 ± 17.09	71.45 ± 12.60	69.90 ± 14.44	68.70 ± 13.84	0.001*	***P*** **-value** ^d^	***P*** **-value** ^f^
	**GA**	77.48 ± 11.40	68.00 ± 12.02	68.23 ± 12.26	68.77 ± 15.52	70.68 ± 15.87	68.73 ± 13.87	65.64 ± 9.45	<0.001*	<0.001*	0.109
*P*-value^a^		0.413	0.027*	0.080	0.565	0.645	0.958	0.566			
MAP (mmHg)	**SPA**	97.67 ± 14.88	93.14 ± 14.07	89.86 ± 11.56	88.81 ± 14.13	85.67 ± 15.19	87.29 ± 11.51	87.57 ± 12.50	0.001*	0.582	0.032*
	**PB**	97.80 ± 13.23	94.10 ± 12.53	92.15 ± 12.12	88.65 ± 17.34	85.95 ± 13.92	85.95 ± 14.92	84.05 ± 14.15	<0.001*	***P*** **-value** ^d^	***P*** **-value** ^f^
	**GA**	93.71 ± 11.53	84.95 ± 12.69	85.86 ± 13.12	85.95 ± 15.30	88.36 ± 16.33	85.86 ± 14.27	82.23 ± 10.17	<0.001*	<0.001*	0.091
*P*-value^a^		0.533	0.050	0.251	0.798	0.816	0.931	0.359			
HR (beats per minute)	**SPA**	84.48 ± 12.83	87.67 ± 9.25	81.95 ± 11.16	84.10 ± 11.01	82.33 ± 14.38	81.10 ± 13.49	81.05 ± 14.13	0.229	0.037*	0.444
	**PB**	86.85 ± 11.82	85.45 ± 9.73	78.50 ± 9.38	81.25 ± 11.72	77.90 ± 9.99	77.45 ± 9.86	75.30 ± 10.36	0.003*	***P*** **-value** ^d^	***P*** **-value** ^f^
	**GA**	82.95 ± 13.28	80.86 ± 12.78	76.55 ± 11.91	78.95 ± 12.01	82.27 ± 15.81	78.41 ± 11.46	75.95 ± 10.39	<0.001*	<0.001*	0.380
*P*-value^a^		0.15	0.115	0.354	0.268	0.499	0.587	0.233			
SpO_2_ (mmHg)	**SPA**	96.71 ± 3.55	97.57 ± 2.18	97.67 ± 2.24	97.62 ± 2.20	97.71 ± 2.10	97.90 ± 2.07	97.71 ± 2.26	0.260	0.002*	0.290
	**PB**	96.60 ± 1.93	97.45 ± 1.70	97.20 ± 1.98	98.05 ± 1.14	97.60 ± 1.84	98.10 ± 1.61	97.75 ± 1.74	0.018*	***P*** **-value** ^d^	***P*** **-value** ^f^
	**GA**	98.67 ± 0.96	99.27 ± 0.70	99.27 ± 0.70	98.95 ± 1.17	98.91 ± 1.065	98.95 ± 0.899	98.95 ± 1.04	0.001*	<0.001*	0.310
*P*-value^a^		0.011*	0.001*	0.001*	0.024*	0.027*	0.079	0.076			
Sedation levels	**SPA**	1.00 ± 0.01	1.29 ± 0.46	1.95 ± 0.29	2.14 ± 0.47	2.14 ± 0.47	2.10 ± 0.46	2.10 ± 0.43	<0.001*	<0.001*	<0.001*
	**PB**	1.00 ± 0.01	1.90 ± 0.31	1.95 ± 0.39	1.95 ± 0.39	2.00 ± 0.32	2.00 ± 0.32	2.00 ± 0.32	<0.001*	***P*** **-value** ^d^	***P*** **-value** ^f^
	**GA**	1.00 ± 0.01	4.00 ± 0.01	4.00 ± 0.01	4.00 ± 0.01	4.00 ± 0.01	4.00 ± 0.01	4.00 ± 0.01	0.309	<0.001*	<0.001*
*P*-value^a^		–	0.001*	0.001*	0.001*	0.001*	0.001*	0.001*			
VAS score	**SPA**	0.00	0.00	0.14 ± 0.65	0.05 ± 0.22	0.05 ± 0.22	0.05 ± 0.218	0.05 ± 0.218	0.329	<0.001*
	**PB**	0.00	1.80 ± 1.79	2.65 ± 1.31	2.75 ± 1.12	1.65 ± 1.14	1.20 ± 1.436	0.75 ± 1.372	0.024*	***P*** **-value** ^d^	***P*** **-value** ^f^
*P*-value^a^		0.001*	0.001*	0.001*	0.001*	0.001*	0.001*	<0.001*			

SPA, spinal anesthesia; PB, paracervical block; GA, general anesthesia; SBP, systolic blood pressure; DBP, diastolic blood pressure; MAP, mean arterial pressure; HR, heart rate; SpO_2_, oxygen saturation; VAS, visual analog scale for pain. *Bold: *P* < 0.05 was considered as significant. ^a^*P*-value based on ANCOVA between three groups. ^b^*P*-value based on paired *t*-test within group. ^c^Tests between groups effects based on two-way analysis of variance with repeated measures (RMANOVA). ^d^Time main effect based RMANOVA. ^e^Unadjusted the interaction effect of group and time (time × group) based on RMANOVA; adjusted (adjusting for age, weight, gravida, and abortion) the time × group effect based on RMANOVA. ^f^Adjusted p-value the time * group effect based on RMANOV.

**FIGURE 4:**
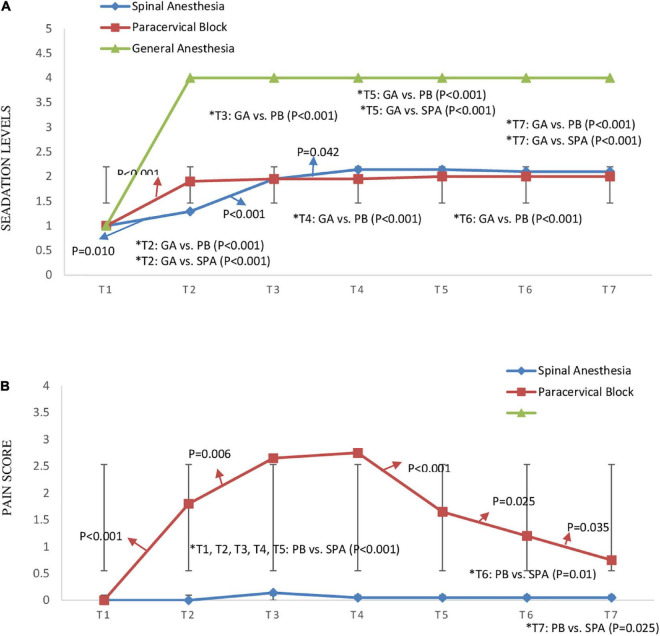
Time trend changes in panel **(A)** sedation levels based on “Ramsay Sedation Scale” between three groups of study and **(B)** pain score based on visual analog scale (VAS) between spinal anesthesia and paracervical block groups (pain score was not measured for general anesthesia). Time trends as follows; T1: pre-anesthesia, T2: during-anesthesia, T3: during-tenaculum, T4: during-cervical dilation, T5: during-hysteroscopy, T6: during-biopsy, and T7: during-hysteroscopy removal. The *P*-value within group differences are indicated by arrows and between groups by *. SPA, spinal anesthesia; PB, paracervical block; GA, general anesthesia.

The monitoring of the sedation levels in the three groups from pre-anesthesia until hysteroscopy removal showed that sedation was significantly greater in the GA group compared to the SPA (Figure 4A) and PB groups, at anesthesia, tenaculum insertion, cervical dilation, hysteroscopy, biopsy, and hysteroscopy removal, with no difference observed between the SPA and PB group (*P* > 0.05) (Figure 4B). In contrast, the VAS score of the PB group was significantly higher than the SPA group at anesthesia (1.80 ± 1.795 vs. 0, *P* = 0.001), tenaculum insertion (2.65 ± 1.309 vs. 0.14 ± 0.655, *P* < 0.001), cervical dilation (2.75 ± 1.118 vs. 0.05 ± 0.218, *P* < 0.001), hysteroscopy (1.65 ± 1.137 vs. 0.05 ± 0.218, *P* < 0.001), biopsy (1.20 ± 1.436 vs. 0.05 ± 0.218, *P* = 0.01), and removal hysteroscope (0.75 ± 1.372 vs. 0.05 ± 0.218, *P* = 0.025) (Figure 4B).

### Comparison of complications and patient satisfaction

Hypotension, bradycardia, shivering, pain, sedation levels, nausea/vomiting, analgesic requirement and patient satisfaction scores were compared between the three groups of study and the results are presented in Table 3. According to our findings, the mean pain score during recovery was found to be significantly higher in the GA group than that of the SPA group (3.77 ± 2.25 vs. 0.10 ± 0.30, *P* < 0.001) and the PB group (3.77 ± 2.25 vs. 0.90 ± 1.37, *P* < 0.001). However, no statistically significant difference was observed between the mean pain scores of SPA and PB groups (0.10 ± 0.30 vs. 0.90 ± 1.3, *P* = 0.661). The requirement for analgesic injections after surgery was also significantly higher in the GA group compared to the PB (50 vs. 10%, *P* < 0.001) and SPA (50 vs. 0%, *P* < 0.001) groups. In terms of sedation levels, the frequency of patients who were more conscious and co-operative was significantly lower in the GA groups compared to the SPA (31.8 vs. 71.4%, *P* = 0.002) and PB groups (31.8 vs. 90%, *P* < 0.001). However, there were no significant differences between three groups according to incidence of hypotension (*P* = 0.157), bradycardia (*P* = 0.521), shivering during surgery (*P* = 0.058), shivering during recovery (*P* = 0.110), nausea/vomiting (*P* = 0.382), and vertigo (*P* = 0.370). The satisfaction level in the patients of GA group was higher than the other two groups, with 82% of patients reporting excellent satisfaction with their operation under GA, compared to 52 and 70% in SPA and PB groups, respectively, although this difference was not statistically significant (*p* = 0.334).

**TABLE 3:** Comparison of complications (during surgery and recovery), sedation level and satisfaction scores among three study groups.

Variables		Groups (*n* = 63)			*P*-value
		SPA (*n* = 21)	PB (*n* = 20)	GA (*n* = 22)	
**During surgery**					
Hypotension	Yes (%)	3 (14.3)	0	1 (4.5)	0.157^a^
Bradycardia	Yes (%)	1 (4.8)	3 (15)	3 (13.6)	0.521^a^
Shivering	Yes (%)	4 (19)	1 (5)	0	0.058^a^
**During recovery**					
Shivering	Yes (%)	7 (33.3)	3 (15)	2 (9.1)	0.110^a^
Intensity of shivering	Median (IQR)	4 (3–4)	2 (1–2)	4 (3–4)	0.241^b^
Pain	Mean ± SD	0.10 ± 0.30	0.90 ± 1.37	3.77 ± 2.25	<**0.001**^c^*
Categorized of pain	No pain (%)	19 (90.5)	12 (60)	6 (27.3)	<**0.001**^a^*
	Mild pain (%)	2 (9.5)	6 (30)	3 (13.6)	
	Moderate pain (%)	0	2 (10)	7 (31.8)	
	Severe pain (%)	0	0	6 (27.3)	
Sedation levels	Anxious and agitated or restless or both (%)	14 (19)	2 (10)	8 (36.4)	**0.002** ^a^ *
	Co-operative, oriented and tranquil (%)	15 (71.4)	18 (90)	7 (31.8)	
	Responding to command (%)	2 (9.5)	0	7 (31.8)	
Nausea and vomiting	Yes (%)	2 (9.5)	1 (5)	4 (18.2)	0.382^a^
Vertigo	Yes (%)	2 (9.5)	0	2 (9.1)	0.370^a^
Analgesic requirement	Yes (%)	0	2 (10)	11 (50)	<0.001*
Satisfaction scores	Excellent (%)	11 (52.4)	14 (70)	18 (81.8)	0.334^a^
	Satisfactory (%)	4 (19)	3 (15)	2 (9.1)	
	Fair (%)	6 (28.6)	3 (15)	2 (9.1)	
	Unsatisfactory (%)	0	0	0	

SPA, spinal anesthesia; PB, paracervical block; GA, general anesthesia.

^a^Chi-square test.

^b^Kruskal-Wallis test.

^c^One-way ANOVA (followed by *post-hoc* Bonferroni’s test).

*Bold: *P* < 0.05 considered as significant, pain was assessed based on VAS score, sedation was assessed by Ramsay scale.

### Logistic regression findings

**FIGURE 5:**
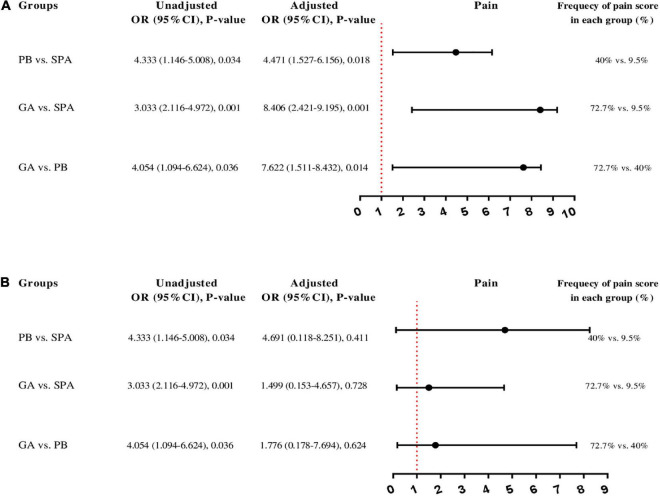
Unadjusted and adjusted binary logistic regression analysis to predict pain score based on visual analog scale (VAS) between the three groups 0 of study; **(A)** the OR adjusting for age, weight, gravid, abortion, and cause of hysterectomy and forest blot figure shows adjusted OR, **(B)** the OR adjusting for surgery duration, anesthesia duration, return time of motor function and recovery duration and forest blot figure shows adjusted OR. In addition, the percent shows the frequency of pain score in each group. SPA, spinal anesthesia; PB, paracervical block; GA, general anesthesia; OR, odds ratio; CI, confidence interval.

Unadjusted binary logistic regression analysis showed that the risk of pain based on VAS score during recovery was significantly increased in the PB (OR: 4.333, 95% CI: 1.146–5.008, *P* = 0.034) and GA (OR: 3.033, 95% CI: 2.116–4.972, *P* < 0.001) groups compared with the SPA group. In addition, the risk of pain score was significantly higher in the GA group (OR: 4.054, 95% CI: 1.094–6.624, *P* = 0.036) than the PB group (Figure 5). In adjusted regression analysis (adjusting for age, weight, gravid, abortion, and cause of hysterectomy), the OR of pain score during recovery was increased in PB (OR: 4.471, 95% CI: 1.527–6.156, *P* = 0.018) and GA (OR: 8.406, 95% CI: 2.421–9.195, *P* = 0.001) groups compared with SPA group (Figure 5A). However, in adjusting based on surgery duration, anesthesia duration, return time of motor function and recovery duration, the ORs of pain score between groups was not statistically significant; PB vs. SPA (OR: 4.691, 95% CI: 0.118–8.251, *P* = 0.411), GA vs. SPA (OR: 1.499, 95% CI: 0.153–4.657, *P* = 0.728), and GA vs. PB (OR: 1.776, 95% CI: 0.178–7.694, *P* = 0.624) (Figure 5B).

## Discussion

The rate of recovery in outpatient hysteroscopy is one of the most important factors related to the patient’s discharge, which largely depends on the method of anesthesia (3). Despite the fact that many studies have been conducted regarding the safest and most cost-effectiveness method of pain reduction in diagnostic hysteroscopy (39, 40), no study has been performed comparing the three methods of SPA, PB, and GA, and there is still an open field to be explored. This study aimed to compare SPA, PB, and GA, in terms of pain intensity, the frequency of nausea and vomiting and analgesic requirements after diagnostic hysteroscopy. According to the result of the present study, the mean pain score (based on VAS score) in recovery and the need for analgesic injections was found to be significantly higher in the GA group compared to that in the SPA and PB groups. While, there was no significant difference between SPA and PB groups in terms of pain score and need for analgesia after surgery, during surgery, the pain score in the PB group was significantly higher than that in the SPA group.

In a similar study, Hosseinzadeh et al. (18), reported significantly higher post-operative pain, and morphine requirement during recovery and 6 and 12 h after surgery in patients undergoing hysterectomy under the GA compared to SPA. Moreover, Massicotte et al. (6), reported significantly higher post-operative pain in the GA group compared to that in the SPA group. In the present study, the intensity of pain and analgesic requirements during recovery, was higher in the GA group than the SPA group. It is consistent with the results of Hosseinzadeh et al.’s and Massicotte et al. ’s studies. In a recent study by Mortazavi et al. (41), in a cross-sectional descriptive study in 350 patients under either SPA or GA for abdominal hysterectomy showed that the recovery quality in GA was lower in comparison with SPA. Carli et al. (42), indicate that in patients underwent abdominal hysterectomy who received SPA combined with sedation considered quality of post-operative recovery better than the patients who received EDA combined with GA. The results of these two studies confirm the results of the present study. Naghibi et al. (43), found less pain in the SA group only within the first 4 h after lower abdominal surgery, while no significant differences were observed afterward. In a study by Junttila et al. (44), comparing paracervical and spinal block for labor analgesia, the results showed that the median pain scores decreased significantly in both groups, although this was greater in the SPA. As in the present study, the paracervical group had a higher pain score than the spinal group during the procedure. In addition, a study by Manninen et al. (45), was conducted on 44 healthy primiparous parturients who randomized receive either PB or epidural EDA. The results showed that the both methods provided in general good analgesia, but the need of analgesia was required more often after PB. The results of this study, like the present study, showed that patients in the PB group had a higher pain score than the regional group.

In the current study, the intensity of sedation was significantly deeper in the GA group compare with the PB and SPA groups during recovery. However, there was no significant difference between the PB group and SPA group in terms of sedation levels. In addition, no significant differences were observed between three groups of the study, in terms of hypotension, bradycardia, shivering during surgery and recovery, ephedrine and atropine administered, post-operative nausea and vomiting, vertigo or satisfaction of anesthesia. In this regard, the results of similar studies reported no significant differences between the SPA and GA groups in terms of nausea and vomiting after surgery (46, 47). In the study of Wallage et al. (48), 191 women candidates for endometrial resection were performed in two groups under GA and local anesthesia (PB). There was no significant difference between the two groups in terms of recovery time, nausea and vomiting which confirms the results of the present study. On the other hand, some previous studies were not in accordance with the results of this study, and showed that the frequency of nausea/vomiting was greater in the GA group compared to the SPA group (18, 41). Most of the drugs used in GA can cause nausea and vomiting, but this difference can be due to the use of propofol in induction of anesthesia in the present study. Nausea and vomiting in surgeries (e.g., hysterectomy) can put pressure on the intestine and the lower gastrointestinal tract, which can have adverse effects on the results of hysterectomy (41). Therefore, the type of anesthesia method should be considered by anesthesiologists to minimize nausea and vomiting.

According to our findings, the use of the SPA method significantly reduces the severity of pain in the recovery unit, leading to a reduction in the need for analgesics. Pain is one of the main indicators of the quality of recovery after surgery and there is an inverse relationship between the severity of pain and the quality of recovery so that more severe pain results in lower quality of recovery (49, 50). The results of this study showed that despite lower pain during recovery in the SPA group compared to the GA group, anesthesia duration, recovery period, and return of motor function were found to the significantly longer in this group compared to the PB and GA groups. The prolonged return of motor function after SPA is because a maximum of 4 sensory dermatomes regress every hour (51, 52). Therefore, it seems that PB with less recovery time and faster return of motor function than SPA and also mild pain during recovery compared to GA can be a good option for hysteroscopy.

To our best knowledge, this study was the first study in this field exploring the effect of three types of anesthesia (SPA, GA, and PB) on the outcomes and complications of diagnostic hysteroscopy surgery. However, this study had some limitations. First, since the anesthesia method must be chosen according to the patient’s wishes, the allocation of patients to three study groups was non-random and the study was single-blinded to the collectors/analyzers and the patient and the anesthesiologist were aware of the type of anesthesia. Secondly, the small number of samples in each group and three cases of failure in the paracervical and spinal groups, thirdly, the use of sedative drugs such as midazolam and fentanyl in regional procedures, which could have an impact on their outcomes, and finally, the lack of monitoring of the hemodynamic status and evaluation of the pain score after discharge from the recovery unit, which can affect the quality of recovery in the first 24 h, were other limitations of this research.

## Conclusion

The present study showed that SPA and PB are able to reduce pain during acute recovery after diagnostic hysteroscopy and can be reasonable substitutes for GA. However, each these methods have its own advantages and disadvantages. Despite lower pain reported in the SPA group compared with the PB group, surgery and recovery duration, as well as return of motor function were longer in patients receiving SPA. Further randomized controlled studies with larger sample sizes are required to determine whether SPA and PBs should be considered in more patients undergoing diagnostic hysteroscopy.

## Data availability statement

The original contributions presented in this study are included in the article/Supplementary material, further inquiries can be directed to the corresponding author.

## Ethics statement

The studies involving human participants were reviewed and approved by the Hamadan University of Medical Sciences (IR.UMSHA.REC.1399.875). The patients/participants provided their written informed consent to participate in this study.

## Author contributions

FR-B, NM, FE-A, and SM: study concept and design. FR-B, NM, and SP: analysis and interpretation of data. NM: acquisition of data and drafting of the manuscript. FR-B, NM, and FE-A: critical revision of the manuscript for important intellectual content. SM and SP: statistical analysis. All authors contributed to the article and approved the submitted version.

## Footnotes

1
http://www.irct.ir
