# Supplementary material for: Comparison of the effects of spinal anesthesia, paracervical block and general anesthesia on pain, nausea and vomiting, and analgesic requirements in diagnostic hysteroscopy: A non-randomized clinical trial
Source: Front Med (Lausanne). 2023 Mar 1;10:1089497. doi: 10.3389/fmed.2023.1089497 (PMC10016381; doi:10.3389/fmed.2023.1089497)
Supplement: Supplementary file 1 [file Table_1.docx]

**Table S1:** Measurement tools that were used in this study

| Tools | **Used for** | **Descriptions** |
| --- | --- | --- |
| Visual Analogue Scale (VAS) | Pain intensity | The VAS consists of a 10 cm line, with two end points representing 0 “no pain” and 10 “pain as bad as it could possibly be” which includes;  0 “no pain”  <3 “Mild pain”  ≥3-6 “Moderate pain”  7-10 “Severe pain” |
| Ramsay Sedation Scale | Sedation rate | It divides a patient's level of sedation into six categories ranging from severe agitation to deep coma which includes;  1 “Anxious and agitated or restless or both”  2 “Co-operative, oriented and tranquil”  3 “Responding to commands only”  4 “Brisk response to light glabellar tap or loud auditory stimulus”  5 “Sluggish response to light glabellar tap or loud auditory stimulus”  6 “No response to stimulus” |
| Bromage scale | measurement of motor block | The modified Bromage Scale categorizes motor block into four categories as follows:  0 "No motor block: free movement of legs and feet"  1 "Partial motor block (33%): able to flex knee free movement of feet, unable to raise extended leg"  2 "Almost complete motor block (66%): free movement of feet only”  3 "Complete motor block: unable to move hips, knees and feet" |
| Modified Aldrete Score | Discharge criteria | ***Activity:***  Able to move 4 extremities (2 points)  Able to move 2 extremities (1 points)  Unable to move extremities (0 points) |
|  |  | ***Respiration:***  Able to breathe deeply and cough freely (2 points)  Dyspnea or limited breathing (1 points)  Apneic (0 points) |
|  |  | ***Circulation:***  BP +/- 20% of pre-anesthetic level (2 points)  BP +/- 20-49% of pre-anesthetic level (1 points)  BP +/- 50% of pre-anesthetic level (0 points) |
|  |  | ***Consciousness:***  Fully awake (2 points)  Arousable on calling (1 points)  Not responding (0 points) |
|  |  | ***Oxygen saturation:***  Able to maintain O2 saturation >92% on room air (2 points)  Needs oxygen to maintain O2 saturation >90% (1 points)  O2 saturation <90% even with supplemental oxygen (0 points) |
